# Expression, purification and characterization of soluble red rooster laforin as a fusion protein in *Escherichia coli*

**DOI:** 10.1186/1471-2091-15-8

**Published:** 2014-04-02

**Authors:** M Kathryn Brewer, Satrio Husodo, Vikas V Dukhande, Mary Beth Johnson, Matthew S Gentry

**Affiliations:** 1 Department of Molecular and Cellular Biochemistry and Center for Structural Biology, College of Medicine, University of Kentucky, 741 S. Limestone, Lexington, Kentucky 40536-0509, USA

**Keywords:** Laforin, Lafora disease, Phosphatase, Carbohydrate-binding module, Glycogen

## Abstract

**Background:**

The gene that encodes laforin, a dual-specificity phosphatase with a carbohydrate-binding module, is mutated in Lafora disease (LD). LD is an autosomal recessive, fatal progressive myoclonus epilepsy characterized by the intracellular buildup of insoluble, hyperphosphorylated glycogen-like particles, called Lafora bodies. Laforin dephosphorylates glycogen and other glucans *in vitro*, but the structural basis of its activity remains unknown. Recombinant human laforin when expressed in and purified from *E. coli* is largely insoluble and prone to aggregation and precipitation. Identification of a laforin ortholog that is more soluble and stable *in vitro* would circumvent this issue.

**Results:**

In this study, we cloned multiple laforin orthologs, established a purification scheme for each, and tested their solubility and stability. *Gallus gallus* (Gg) laforin is more stable *in vitro* than human laforin, Gg-laforin is largely monomeric, and it possesses carbohydrate binding and phosphatase activity similar to human laforin.

**Conclusions:**

Gg-laforin is more soluble and stable than human laforin *in vitro*, and possesses similar activity as a glucan phosphatase. Therefore, it can be used to model human laforin in structure-function studies. We have established a protocol for purifying recombinant Gg-laforin in sufficient quantity for crystallographic and other biophysical analyses, in order to better understand the function of laforin and define the molecular mechanisms of Lafora disease.

## Background

Lafora disease (LD) is an autosomal recessive, neurodegenerative disorder resulting in myoclonus, epilepsy, dementia, and death [1-3]. Affected individuals experience an initial seizure during adolescence, followed by severe neurological decline until the patient’s death approximately ten years after the first seizure [1,4]. Characteristic of the disease is the cytoplasmic accumulation of hyperphosphorylated glycogen-like particles called Lafora bodies (LBs) in various tissues including brain, muscle and liver [1,5].

Approximately 50% of Lafora disease cases are caused by mutations in the *EPM2A* (epilepsy of progressive myoclonus type 2 gene A) gene that encodes the protein laforin [4-6]. *EPM2A* is conserved in all vertebrate genomes, but it is absent from the genome of most non-vertebrate organisms including standard model organisms such as *Saccharomyces cerevisiae*, *Caenorhabditis elegans*, and *Drosophila melanogaster*[7,8]. An exception to this rule is a small subgroup of protists that synthesize floridean starch, an insoluble carbohydrate similar to LBs. Five protozoan laforin orthologs have been identified; however, sequence identity between these proteins and human laforin is <37% and the genes have major insertions and deletions [7,8]. Thus, these proteins are not optimal orthologs to utilize for modeling human laforin.

Laforin is a bimodular protein with a carbohydrate-binding module (CBM) at its amino-terminus and a dual-specificity phosphatase (DSP) domain at its carboxy-terminus [9-11]. CBMs are most commonly found in glycosyl hydrolases and glucosyl transferases from bacteria, fungi or plants, and there are over 39 families of CBMs that bind a variety of carbohydrate substrates. Laforin belongs to the CBM20 family according to the Carbohydrate-Active Enzymes (CAZy; http://www.cazy.org) database [12]. CBM20s are closely related to CBM48s, and both are classified as starch-binding domains with similar folds and binding sites [13-15]. Typical of DSPs, laforin is capable of hydrolyzing phosphotyrosine and phosphoserine/phosphothreonine substrates; however, laforin is unique among phosphatases in that it is the only phosphatase in humans containing a CBM, which targets laforin to glycogen [8,9]. Laforin has been shown to bind and dephosphorylate glycogen and other glucans *in vitro* and *in vivo*[8,9,16-19].

Glycogen is an energy storage molecule synthesized by bacterial, fungal and animal species consisting of α-1,4 and α-1,6 linked residues of glucose, with 12-14 residues per branch [20]. Glycogen has been shown to contain small amounts of phosphate, but the regulation and effects of this phosphorylation event are currently under debate [19,21-25]. While the source of phosphorylation is disputable, data from multiple labs has clearly established that loss of laforin activity results in hyperphosphorylation and poorly branched glycogen, resulting in insoluble LBs [17-19,23,26].

Although the substrate and function of laforin have recently been elucidated, the structural basis for the unique glucan phosphatase activity of laforin remains unknown. Ourselves and others have experienced difficulty purifying laforin in sufficient quantities and of sufficient quality for crystallographic studies [27]. One group recently demonstrated that recombinant human laforin expressed in *E. coli* is largely insoluble and must be purified from inclusion bodies [27]. This procedure requires denaturation and refolding steps, involves harsh chemical treatments, and often yields low amounts of correctly folded protein. A subsequent report demonstrated that only the laforin CBM was soluble when expressed in *E. coli*[28].

Our lab has purified enough recombinant laforin from the soluble portion of bacterial cell lysates to perform *in vitro* assays [8,16,29-31]. However, the protein often aggregates and precipitates after the multistep purification procedure. In this study, we found that the addition of sugars to the lysis and purification buffers increases the yield of soluble laforin from lysates and improves stability. However, such additives interfere with methods such as isothermal titration calorimetry that directly measure protein-ligand interactions. Also, we have been unable to crystallize laforin purified in the presence of sugars (unpublished data). Our group recently determined the structures of two glucan phosphatases from *Arabidopsis* that are functionally similar to laforin, and the structures of other DSP domains and CBMs are available [32,33]. However, these structures provide little information about the function of laforin due to low similarity between these domains and the domains of laforin. We then sought a laforin ortholog that is highly similar to human laforin (Hs-laforin) and, when expressed in bacteria, is less prone to aggregation and precipitation. We cloned and purified multiple laforin orthologs and optimized the purification of recombinant *Gallus gallus* laforin (Gg-laforin). Previously, the CBM of Gg-laforin was fused to a glutathione *S*-transferase (GST) tag and shown to bind glycogen [34]. In this study, we purified SUMO-tagged full-length Gg-laforin and confirmed that Gg-laforin functions as a monomer, contrary to prior claims that laforin dimerization is necessary for phosphatase activity [27,35]. Phosphatase and glucan binding assays indicate that the catalytic and binding ability of Gg-laforin is comparable to that of Hs-laforin [8,30,36]. Therefore, Gg-laforin is an excellent model for Hs-laforin and a better alternative for crystallization and other biophysical studies.

## Results and discussion

### Instability of Hs-laforin and other laforin orthologs

**Figure 1:**
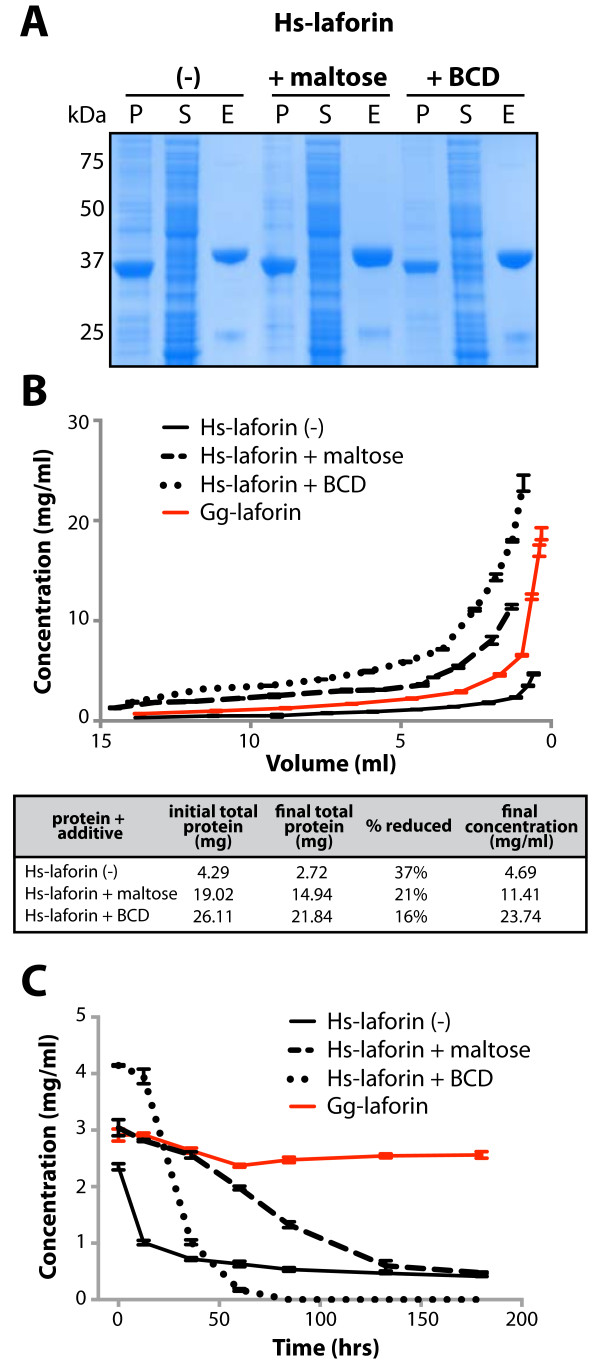
**Purification and stability analysis of Hs-laforin and Gg-laforin. A**. Hs-laforin was expressed in *E. coli* and purified by affinity chromatography in the absence (-) and presence of 15% maltose or 10 mM β-cyclodextrin (BCD). Fractions of the pellet (P) and supernatant (S) after high-speed centrifugation and fractions of the IMAC elution (E) were analyzed by SDS-PAGE and stained with Coomassie Blue dye. **B**. Elution fractions from Hs-laforin and Gg-laforin preparations were concentrated using centrifugal filter units. Volume and concentration of each preparation were monitored throughout centrifugation, and protein concentration was measured using a Bradford assay. Total protein content for each preparation before and after concentration and the percent reduced were calculated for each preparation. **C**. Elution fractions were concentrated to approximately 2-4 mg/ml and incubated at room temperature for eight days. The concentration of each protein was measured during the course of the experiment using a Bradford assay.

Soluble Hs-laforin has proved to be a difficult protein to purify from *E. coli*[27,28]. While we have successfully purified some Hs-laforin suitable for *in vitro* assays, the protein is unstable and precipitates from solution. Thus, we sought to optimize the purification procedure using an additive. His_6_-tagged Hs-laforin was expressed and purified from *E. coli* by affinity chromatography. Approximately 5 mg of soluble Hs-laforin was obtained from 1 L of *E. coli* cells. In order to increase the solubility of Hs-laforin, we tested the addition of the sugars maltose and β-cyclodextrin (BCD) to the purification buffer. The addition of 15% maltose (w/v) or 10 mM BCD to the lysis and purification buffers improved the yield of soluble Hs-laforin to 8 mg and 9 mg per 1 L culture, respectively (Figure 1A). Next we sought to define the stability of recombinant Hs-laforin purified in the different buffers using two methods.

We first determined the stability of Hs-laforin by concentrating the protein using centrifugal filter units and measuring the volume and concentration throughout the centrifugation process. The Hs-laforin preparation without added sugars did not exceed 5 mg/ml and total soluble protein was reduced by 37% during the centrifugation process (Figure 1B). Conversely, Hs-laforin purified in the presence of maltose or BCD was concentrated to >11 mg/ml, and total soluble protein content was reduced by less than 21% (Figure 1B). Thus, the addition of BCD or maltose allows Hs-laforin to be concentrated to higher concentrations likely by preventing aggregation and precipitation. Second, we sought to define the long-term stability of Hs-laforin +/- sugars. Hs-laforin was incubated at room temperature and protein concentrations were measured over a period of eight days. After only 12 hours, the concentration of Hs-laforin had fallen significantly and continued to drop over the eight-day period (Figure 1C). With the addition of maltose, the concentration did not decrease as rapidly, confirming that the addition of maltose improves the stability of laforin over long periods of time. The addition of BCD improved the stability of laforin in the first 12 hours, but subsequently the concentration rapidly decreased and Hs-laforin in the presence of BCD became completely insoluble after 85 hours. Crystallography often demands that proteins be stable at high concentrations and for extended periods of time. These data demonstrate that the addition of BCD or maltose inhibits Hs-laforin from precipitating. While these results represent an improvement over previously reported Hs-laforin purification strategies, crystallization trials in our lab have demonstrated that the presence of BCD or maltose inhibits Hs-laforin crystallization, possibly due to increased heterogeneity in the sample (unpublished data).

**Figure 2:**
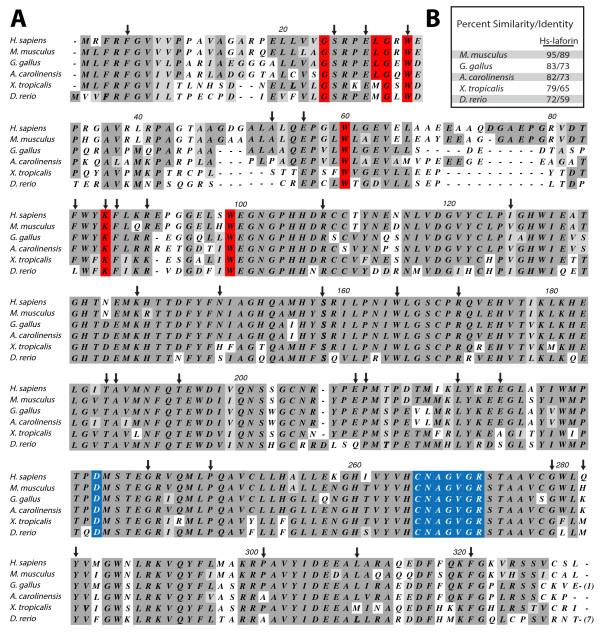
**Comparison of Hs-laforin with laforin orthologs from five vertebrate species. A**. Amino acid sequence alignment of Hs-laforin and five laforin orthologs. Dark grey indicates identical residues, while similar residues are highlighted in light grey. Resides in red boxes are invariant residues as defined by the CBM20 family [15]; blue boxes indicate residues that are part of the phosphatase catalytic site. Locations of 42 missense mutations in Hs-laforin associated with LD are marked with arrows. The numbering of amino acids corresponds to the Hs-laforin sequence. **B**. Percent similarity and identity of Hs-laforin compared with laforin orthologs from other vertebrates.

While the addition of maltose or BCD increases the stability of Hs-laforin, in addition to inhibiting crystallization, the presence of a sugar additive would interfere with glucan binding experiments and other biophysical assays. Therefore, we set out to identify a laforin ortholog that is similar to Hs-laforin, but more stable *in vitro*. Sequences of Hs-laforin and laforin orthologs from *Mus musculus* (mouse), *Gallus gallus* (red rooster), *Xenopus tropicalis* (frog), *Anolis carolinensis* (lizard) and *Danio rerio* (zebrafish) were aligned using ClustalW. Each of these orthologs contains the four invariant aromatic residues characteristic of a laforin CBM (Hs-laforin F5, W32, W60, and W99) and the signature DSP amino acid sequence, DX_30_CX_2_GX_2_R (Figure 2A). Additionally, these orthologs are 72-95% similar to Hs-laforin at the amino acid level (Figure 2B).

We obtained cDNA clones of the *EPM2A* gene from *Mus musculus* (Mm-laforin), *Xenopus tropicalis* (Xt-laforin), and *Gallus gallus* (Gg-laforin). Recombinant Mm-laforin was expressed with a His_6_-tag, and Xt-laforin was expressed as a His_6_-SUMO fusion protein in *E. coli*. Mm-laforin and Xt-laforin were purified in the absence of any sugars and these preparations yielded more soluble protein than Hs-laforin, 6 and 10 mg/L of *E. coli*, respectively. However, the yield for Mm-laforin was not significantly greater than Hs-laforin, and Xt-laforin exhibited the same tendency as Hs-laforin to aggregate and precipitate (Additional file 1: Figure S1). Gg-laforin was also expressed as a His_6_-SUMO fusion protein and purified in the absence of any sugars. Gg-laforin purifications yielded approximately 14 mg/L of *E. coli*, a vast improvement compared to Hs-laforin. We then investigated the *in vitro* stability of recombinant Gg-laforin using the same assays as described for Hs-laforin. We found that Gg-laforin in the absence of any additive can be concentrated to over 18 mg/ml, and the protein is stable >180 hours (Figure 1B-C). Thus, Gg-laforin is much less prone to precipitation compared to Hs-laforin at high concentrations and over long periods, and is more favorable for use in downstream biophysical methods.

### Gg-laforin purification yields a monomeric species

Given recent reports that full-length Hs-laforin cannot be purified as a soluble protein and our data demonstrating its instability, we sought to optimize Gg-laforin purification and to test its biochemical properties to determine whether Gg-laforin would be a good alternative for solving the laforin structure [27,28]. Recombinant His_6_-SUMO-Gg-laforin was expressed and purified from *E. coli* by affinity chromatography, digested with ULP1 to cleave the His_6_-SUMO tag, and subjected to reverse affinity chromatography to remove the tag and His_6_-tagged ULP1. These steps yielded ~10 mg of untagged Gg-laforin per L of bacterial culture.

**Figure 3:**
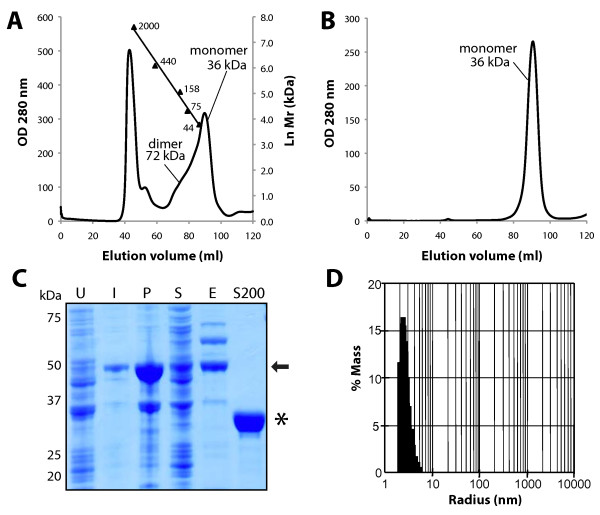
**Purification and characterization of Gg-laforin. A**. The chromatogram is of His_6_-SUMO- tagged Gg-laforin first purified using an IMAC affinity column, and then passed over a HiLoad 16/60 Superdex 200 size exclusion column. Calibration of molecular weight markers is indicated, and ordinates indicate the natural logarithm of molecular weight (Mr). **B**. The monomer fractions were collected, concentrated, and re-loaded over the same column. The chromatogram shows the results of this second round of size-exclusion chromatography. **C**. SDS-PAGE stained with Coomassie Blue of fractions from *E. coli* cells: (U) uninduced cells; (I) induced with IPTG; (P) pelleted/insoluble fraction; (S) soluble fraction; (E) IMAC eluate; and (S200) monomer fraction from Superdex 200 elution. 20 μg of total protein was loaded per lane. His_6_-SUMO-Gg-laforin runs as a 50 kDa species until removal of the His_6_-SUMO tag after IMAC elution (indicated by the arrow). Untagged Gg-laforin is predicted to be 36 kDa (indicated by the asterisk). **D**. Dynamic light scattering was performed on a 1 mg/ml sample of Superdex 200-purified Gg-laforin monomer using a Protein Solutions DynaPro-99 system. Scattering intensity was measured and presented as a fraction of the total protein mass. A single species was detected with a hydrodynamic radius of 2.68 nm, corresponding to a molecular weight of 31.6±14.5 kDa.

Hs-laforin has a propensity to dimerize and form multimers [8,30]. In addition to a multimer peak, Hs-laforin elutes from size exclusion columns as a second peak with a small shoulder of larger molecular weight [8,30]. The small shoulder contains dimerized Hs-laforin and the major peak to the right of this shoulder is monomeric Hs-laforin [30]. In order to determine whether Gg-laforin also forms higher order species, Gg-laforin was subjected to size exclusion chromatography using a Superdex 200 column. Similar to Hs-laforin, Gg-laforin eluted as multiple peaks with a significant amount of protein in the multimer peak (Figure 3A). The chromatogram for the Gg-laforin elution showed a similar pattern as previously reported for Hs-laforin with both a dimer shoulder (~72 kDa) and a monomer peak (~36 kDa) (Figure 3A). Approximately 5 mg of monomeric Gg-laforin was recovered from the size-exclusion elution fractions. To test if Gg-laforin exists in a dynamic monomer/dimer state, we collected the fractions from the monomer peak, concentrated the fractions, and re-loaded these fractions over the same column. Gg-laforin eluted as a 36 kDa protein, and no dimer shoulder was present during this second purification, suggesting that monomeric Gg-laforin does not convert to a dimer (Figure 3B). The protein content and purity of the Superdex 200 monomeric fraction was assessed by collecting fractions and analyzing them by SDS-PAGE. Gg-laforin purified via this multi-step protocol migrated as a highly pure 36 kDa protein (Figure 3C). Previous studies have shown that Hs-laforin dimers are resistant to SDS denaturation to a small extent, but there was no indication from the gel that a Gg-laforin dimer species was present [30,35]. To further define the size and oligomeric state of Gg-laforin, the Superdex 200-purified Gg-laforin protein was analyzed using dynamic light scattering (DLS). The hydrodynamic radius of the detected species corresponded to a 31.6 ± 14.5 kDa protein, the approximate size of the monomeric Gg-laforin (Figure 3D). Cumulatively, these data demonstrate that Gg-laforin can be cleaved from the His_6_-SUMO fusion tag, monomeric Gg-laforin can be resolved by size-exclusion chromatography, and the monomers remain monomeric during subsequent chromatography steps. Thus, Gg-laforin behaves in a similar manner as previously reported for Hs-laforin [7,8,30].

### Gg-laforin monomer binds glucans

**Figure 4:**
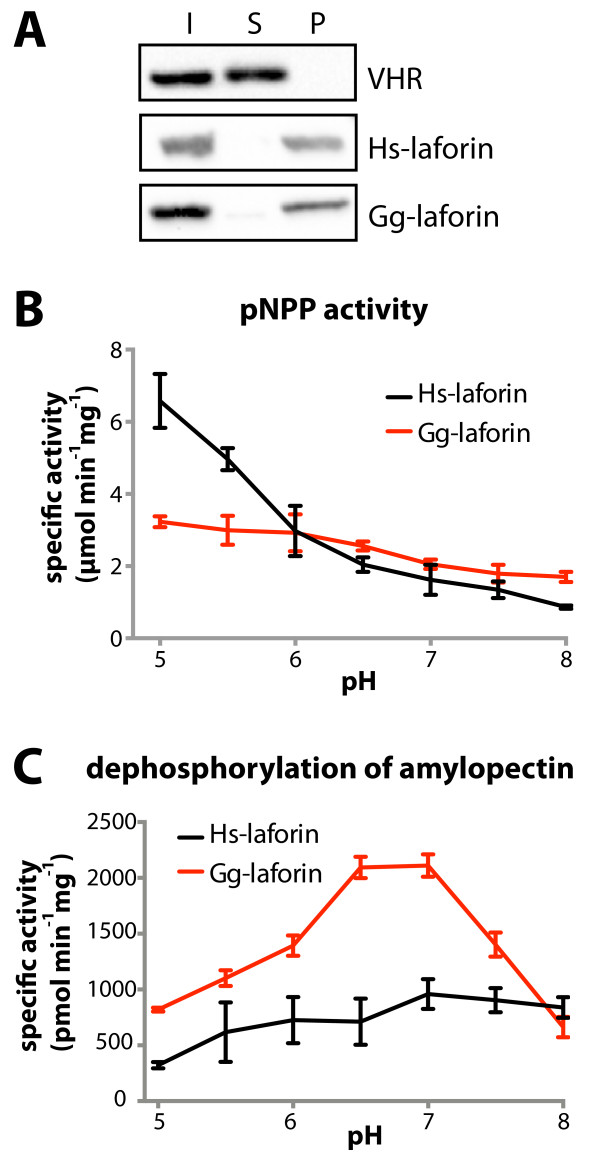
**Analysis of carbohydrate binding and phosphatase activity of Gg-laforin. A**. His-tagged proteins were incubated with amylose resin, then the amylose resin was pelleted by centrifugation, the supernatant was removed, and SDS-PAGE buffer was added to the pellet to release proteins bound to the amylose resin. Proteins in the supernatant were precipitated, and SDS-PAGE buffer was added to the supernatant sample. Protein input (I), supernatant (S) and pellet (P) samples were resolved by SDS-PAGE and visualized by Western analysis. **B**. Specific activities of Gg-laforin and Hs-laforin were quantified against the artificial substrate pNPP at pH units 5.0-8.0. **C**. Phosphate release from amylopectin using Gg-laforin and Hs-laforin at pH units 5.0-8.0 was measured using a malachite green assay. Error bars indicate mean ± SD.

The CBM of Hs-laforin distinguishes this phosphatase from other protein tyrosine phosphatase superfamily members in that the CBM enables Hs-laforin to bind carbohydrates [37]. Gg-laforin is predicted to possess a CBM due to the high similarity between Hs-laforin and Gg-laforin in this region. The CBM of Gg-laforin is highly similar to the Hs-laforin CBM and was previously shown to bind glycogen *in vitro*[34]. Using agarose beads conjugated to the carbohydrate amylose, we investigated the glucan binding properties of Gg-laforin. The Vaccinia H1-related phosphatase (VHR) is a human phosphatase from the same DSP superfamily as laforin, but VHR lacks a CBM and is therefore unable to bind carbohydrates [8]. Hs-laforin, Gg-laforin and VHR were each incubated with amylose beads for 30 min at 4°C, the beads were then pelleted by centrifugation, the supernatant was removed, and the beads were treated with SDS-PAGE buffer to release the proteins bound to the beads. Subsequently, proteins in the supernatant were precipitated and resuspended in SDS-PAGE buffer. Proteins in the supernatant and pellet fractions were separated by SDS-PAGE and analyzed by Western blotting. Gg-laforin bound amylose to the same extent as Hs-laforin, and both were present almost entirely in the pellet (Figure 4A). Alternatively, VHR did not bind the amylose beads and remained in the supernatant as expected (Figure 4A). Thus, Gg-laforin possesses a CBM that is capable of binding amylose to a similar degree as Hs-laforin.

### Gg-laforin monomer has phosphatase activity comparable to Hs-laforin

Another group reported that only Hs-laforin dimers possess phosphatase activity; however, work from our lab and others demonstrated that both monomer and dimer species of Hs-laforin are catalytically active [30,35]. To determine if monomeric Gg-laforin has similar activity as Hs-laforin, monomeric Gg-laforin was assayed for phosphatase activity using the artificial substrate *para*-nitrophenylphosphate (pNPP) over a range of pH values, from 5.0-8.0. Gg-laforin displayed similar specific activity to Hs-laforin and also, like Hs-laforin, displayed a preference for a lower pH (Figure 4B). Mutation of the catalytic cysteine residue (C266) to serine within the DSP of Hs-laforin inactivates the enzyme [8,38]. We cloned and purified a corresponding Gg-laforin C253S mutant, and as expected this mutant displayed no activity and was used as a negative control.

Hs-laforin is the only human phosphatase known to bind and dephosphorylate glycogen and amylopectin *in vitro*[8,16,17]. Therefore, we investigated the ability of Gg-laforin to dephosphorylate the phosphorylated carbohydrate amylopectin using a malachite green-based assay that detects liberated inorganic phosphate [29]. Gg-laforin possesses higher specific activity against phosphorylated amylopectin than Hs-laforin, while preferring a similar pH to Hs-laforin (Figure 4C). These results demonstrate that Gg-laforin is a glucan phosphatase and an ortholog of Hs-laforin, interestingly with a somewhat greater ability to dephosphorylate glucans than Hs-laforin. At the optimal pH, Gg-laforin has a lower specific activity against pNPP. Therefore, the two-fold increase in the specific activity of phosphate release from amylopectin may be due to differences in the CBM of Gg-laforin rather than differences between Hs-laforin and Gg-laforin within the DSP. Indeed, the Hs-laforin and Gg-laforin DSP domains share 84% similarity, while the CBM of Gg-laforin is only 57% similar to the CBM of Hs-laforin [8]. However, most of the amino acids associated with LD mutations are conserved in the Gg-laforin CBM. These data show that Gg-laforin is a glucan phosphatase with similar activity levels as Hs-laforin, yet Gg-laforin is more soluble when purified as a fusion protein in a bacterial expression system.

## Conclusions

Human laforin has proven to be a difficult protein to express in recombinant systems. These difficulties are highlighted by previous reports that Hs-laforin must be purified from inclusion bodies in *E. coli* or that only the Hs-laforin CBM is soluble in *E. coli*[27,28]. While structural information regarding the individual laforin domains would offer some insights into how laforin functions as a glucan phosphatase, the more intriguing questions focus on how the two domains are integrated and how they function synergistically during dephosphorylation of glycogen. Indeed, there are a number of structures of DSP domains and CBMs already determined, but due to the low degree of similarity with the laforin domains they do not offer much insight into the function of laforin [12,39-46]. We recently determined the structure of two *Arabidopsis* glucan phosphatases, Starch EXcess 4 (SEX4) and Like Sex Four 2 (LSF2). SEX4 contains a CBM and DSP domain, while LSF2 lacks a CBM. The individual laforin domains are likely to resemble the domains of SEX4 and LSF2 [32,33]. Indeed, laforin is functionally related to SEX4 and LSF2 (i.e. they are all glucan phosphatases); however, the DSP of laforin is <39% similar to the DSP of SEX4 and LSF2, and the laforin CBM is from an entirely different sub-class of CBM than that of SEX4 [7,8,47-49]. Although SEX4 possesses a CBM and DSP, these domains are in the opposite orientation compared to laforin. SEX4 and LSF2 also each contain a C-terminal motif that integrally folds into the DSP and is essential for maintaining the integrity of the structure [32,33]. Although SEX4 and LSF2 are the first glucan phosphatase structures to be determined, due to multiple differences in domain organization as well as degree of similarity these structures do not offer key insights into the structure of laforin.

Our lab has been successful in purifying sufficient amounts of Hs-laforin for *in vitro* assays without using denaturation and refolding steps, but recombinant Hs-laforin has proved difficult to work with in experiments requiring large quantities of protein, due to low yields and the tendency to aggregate and precipitate. We sought a laforin ortholog with greater solubility and stability yet possessing similar *in vitro* characteristics as Hs-laforin; such an ortholog would be a more conducive target for crystallography and other biophysical techniques. The structure of this ortholog would provide insight into the mechanism of laforin function and may shed light on why mutations in certain amino acids lead to LD.

We have demonstrated that His_6_-SUMO-Gg-laforin is expressed as a soluble protein in *E. coli*, Gg-laforin remains soluble after cleavage of the fusion protein during experimental manipulation, and it possesses both phosphatase and glucan binding activity. Gg-laforin can be purified without the use of denaturation and refolding steps, and the protein does not require a sugar to improve its stability. We showed that Gg-laforin is present as a multimer and monomer, it remains monomeric after size-exclusion chromatography, and it possesses phosphatase and glucan binding activity as a monomer. Monomeric Gg-laforin has robust phosphatase activity against the artificial substrate pNPP and also the more biologically relevant substrate amylopectin, similar to the activity of Hs-laforin as previously described [7,8,30]. Consequently, Gg-laforin is an excellent alternative to Hs-laforin for crystallization trials, and once determined, the structure of Gg-laforin will be a very good model for Hs-laforin in structure-function studies. The characterization of Gg-laforin has provided an alternate route for obtaining the crystal structure of laforin that can be utilized to clarify the role of laforin in the metabolism of insoluble carbohydrates and the etiology of Lafora disease.

## Methods

### Cloning procedures

The ppSUMO plasmid was a generous gift from Dr. Jack Dixon (University of California, San Diego, USA). The plasmid pGL-EPM2A containing the gene for Mm-laforin (NP_034276.2) was a kind gift from Dr. Kazuhiro Yamakawa (Brain Science Institute, Wako-shi, Japan). Mm-laforin was subcloned into pET21a that includes a C-terminal His_6_ tag.

Expressed sequence tags (ESTs) of Xt-laforin (NP_001123695.1) and Gg-laforin (NP_001026240.1) were purchased from Open Biosystems and Delaware Biotechnology Institute, respectively, and cloned into ppSUMO according to standard protocols. ppSUMO encodes a small Ub-like modifier (SUMO) fusion tag that includes an amino-terminal His_6_-tag to aid purification. Sequences were verified by DNA sequencing. pET21a Vaccinia H1-related phosphatase (VHR) and pET21a Hs-laforin constructs have been described previously [8,38].

### Protein expression and purification

All proteins were expressed in BL21-CodonPlus *E. coli* cells (Stratagene) and purified using an IMAC column on a Profinia purification system (BioRad) followed by size exclusion chromatography. Bacterial cultures were grown in 1 L 2xYT or Terrific Broth (IBI Scientific) with 1 mM kanamyacin and 1 mM chloramphenicol at 37°C until OD_600_ reached ~0.8. Cultures were chilled on ice for 20 minutes, and isopropyl thio-β-D-galactopyranoside (IPTG) was added for a final concentration of 0.4 mM to induce protein expression. After growth for approximately 12-16 hours, cells were harvested by centrifugation and stored at -20°C. Bacterial pellets expressing Hs-laforin were resuspended in buffer A: 50 mM Tris/HCl (pH 7.5), 300 mM NaCl, and 2 mM dithiothreitol (DTT). Pellets expressing Mm-laforin were resuspended in buffer B: 50 mM Tris/HCl (pH 8.0), 300 mM NaCl, and 0.05% β-mercaptoethanol. Pellets expressing VHR, Xt-laforin or Gg-laforin were resuspended in buffer C: 20 mM Tris/HCl (pH 7.5), 100 mM NaCl and 2 mM DTT. 15% maltose (w/v) or 10 mM β-cyclodextrin was added to some preparations. Resuspended cells were lysed with a microfluidizer (EmulsiFlex-C5, Avestin), and soluble fractions were separated by high-speed centrifugation (48,000 *g*). His_6_-SUMO-tagged Xt-laforin and Gg-laforin were purified using a Profinia IMAC column (Bio-Rad) with a Profinia protein purification system (Bio-Rad) and dialyzed into buffer C in the presence of the SUMO-specific protease ULP1 that also contains a His_6_-tag. Reverse purification over the Profinia IMAC column was used to remove ULP1-His_6_ and the fusion tag. Each protein was then purified using a HiLoad 16/60 Superdex 200 size exclusion column and ÄKTA FPLC (GE Healthcare). Fractions containing the Gg-laforin monomer species were collected and put back over the same column. Mm-laforin, Hs-laforin and VHR were also expressed as His_6_-tagged recombinant proteins and purified in a similar manner.

### Protein gel electrophoresis, quantitation of stability, and dynamic light scattering

Protein purity was assessed by sodium dodecyl sulfate-polyacrylamide gel electrophoresis (SDS-PAGE). Gels were stained with Coomassie brilliant blue to visualize proteins. To quantify stability of Hs-laforin and Gg-laforin, elution fractions were concentrated using centrifugal filter units (30 K, Amicon Ultra). Volume and concentration were monitored throughout centrifugation at 3,220 × *g*, and protein concentration was measured using a Bradford assay. To test long-term stability, samples were concentrated to approximately 2-4 mg/ml and incubated at room temperature for eight days. Protein concentration was monitored during the course of the experiment using a Bradford assay.

Dynamic light scattering (Protein Solutions DynaPro-99) was utilized to determine the hydrodynamic radius of particles in solution. The DLS system measures the size distribution of particles by detecting fluctuations in light intensity over time. Scattering intensity was presented as a fraction of the total protein mass; poly- or monodispersity in the sample was determined by the number of peaks on the DLS histogram. A standard curve embedded in the DLS software was used to calculate the approximate size of a globular protein with the observed hydrodynamic radius. Measurements were performed on a protein sample of 1 mg/ml at room temperature.

### Glucan binding assay

Amylose immobilized on agarose resin (New England Biolabs) was pre-incubated with 1% BSA at room temperature for 30 min to prevent nonspecific binding. 0.25-1 μg of each recombinant His_6_-tagged protein was mixed with 30 μl amylose beads in buffer C and protease inhibitor cocktail (2.5 mM AEBSF, 2.5 mM benzamidine hydrochloride, 2.5 μM leupeptin, 2.5 μM E64) while rotating at 4°C for 30 min. Amylose beads were pelleted by centrifugation (2,300 × *g*), the supernatant was removed, proteins in the supernatant were precipitated, and proteins in the pellet and supernatant were visualized by Western analysis. Blots were probed with mouse anti-His_6_ 1:4000 (NeuroMabs) and goat anti-mouse HRP (Invitrogen). SuperSignal West Pico (Thermo Scientific) was used to detect the HRP signal.

### Phosphatase assays

Phosphatase activity was determined using the substrates *para*-nitrophenylphosphate (pNPP) and potato amylopectin as described previously [8,29,30]. The pNPP reactions were carried out in 50 μl reactions in 1 × phosphate buffer (0.1 M sodium acetate, 0.05 M bis-Tris, 0.05 M Tris-HCl, and 2 mM DTT at the appropriate pH), 50 mM pNPP, and 200-400 μg enzyme at 37°C for 2 min. Reactions were terminated with the addition of 200 μl 0.25 M NaOH. Absorbance was measured at 410 nm. Malachite green reactions were carried out in 20 μl reactions in 1 × phosphate buffer, 45 μg amylopectin, and 100 ng enzyme at 37°C. After 2-5 minutes, 20 μl 0.1 M *N*-ethylmaleimide and 80 μl malachite green reagent was added to quench the reaction, and absorbances were measured at 620 nm after 40 minutes. Assays were performed in triplicate for each enzyme at pH 5.0, 5.5, 6.0, 6.5, 7.0, 7.5, 8.0.

### Sequence alignment

Amino acid sequences of laforin orthologs were obtained from NCBI, aligned by ClustalW [50], and refined manually using MacVector. LD missense mutations are marked according to listings in the Lafora Progressive Myoclonus Epilepsy Mutation and Polymorphism database (http://projects.tcag.ca/lafora/) [51].

## Abbreviations

LD: Lafora disease; LB: Lafora body; CBM: Carbohydrate-binding module; DSP: Dual-specificity phosphatase; SUMO: Small ubiquitin-like modifier; EST: Expressed sequence tag; VHR: Vaccinia H1-related phosphatase; IPTG: Isopropyl thio-β-D-galactopyranoside; DTT: Dithiothreitol; ULP1: Ubiquitin-like specific protease-1; pNPP: *para*-nitrophenylphosphate; DLS: Dynamic light scattering; BCD: β-cyclodextrin; IMAC: Immobilized metal affinity chromatography; SDS-PAGE: Sodium dodecyl sulfate-polyacrylamide gel electrophoresis.

## Competing interests

The authors declare that they have no competing interests.

## Authors’ contributions

MSG conceived the study. MKB, VVD, MBJ, and MSG carried out the cloning of laforin orthologs. VVD expressed and purified Mm-laforin. MKB and SH carried out expression, purification and characterization of Hs-laforin, Xt-laforin and Gg-laforin. MKB and MSG wrote the manuscript. All authors read and approved the final manuscript.

## Supplementary Material

Additional file 1: Figure S1 Multimerization of Xt-laforin. Xt-laforin was purified by IMAC and passed over a HiLoad 16/60 Superdex 200 size exclusion column. The chromatogram shows a prominent peak corresponding to a multimeric species and unresolved peaks corresponding to the Xt-laforin dimer and monomer (72 kDa and 36 kDa, respectively). Click here for file
