# Supplementary figures and images for: Expression, purification and characterization of soluble red rooster laforin as a fusion protein in Escherichia coli
Source: BMC Biochem. 2014 Apr 2;15:8. doi: 10.1186/1471-2091-15-8 (PMC4234410; doi:10.1186/1471-2091-15-8)

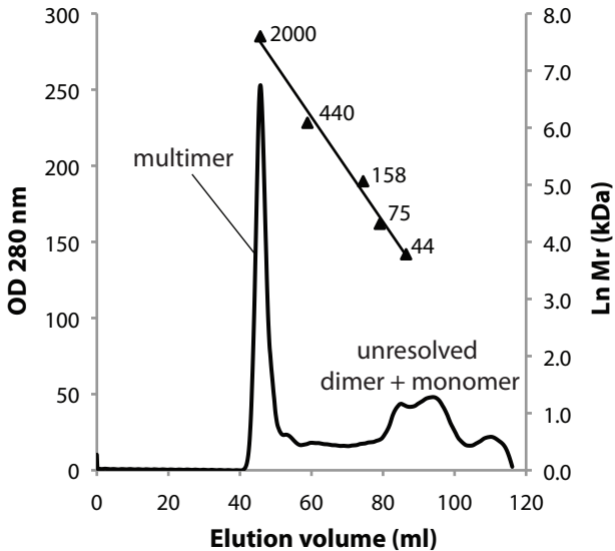

Supplement: Additional file 1: Figure S1 — Multimerization of Xt-laforin. Xt-laforin was purified by IMAC and passed over a HiLoad 16/60 Superdex 200 size exclusion column. The chromatogram shows a prominent peak corresponding to a multimeric species and unresolved peaks corresponding to the Xt-laforin dimer and monomer (72 kDa and 36 kDa, respectively). [file 1471-2091-15-8-S1.pdf]
